# Influence of Terrestial and Atmospheric Electricity upon the System

**Published:** 1847-10

**Authors:** 


4. *Influence of terrestial and atmospheric Electricity upon the human system.*—M. Pallas, principal physician in Algers, presents the results of observations he has recently made in Africa, in order to study the influence of atmospheric and terrestial electricity upon the human system and to modify the pernicious influence of this physical agent by isolation. This work, which is interesting to the etiology, nature and treatment of the diseases of warm climates, may be condensed into the following propositions:

1st. The majority of the diseases, especially those which belong to the class of neuroses, are occasioned by the influence of increased general electricity, of which the thunder clouds and marshy districts are the most abundant sources.

2d. The marshes, by their geographical arrangement and the effects they produce upon the animal economy, present the greatest analogy to the galvanic pile. Indeed their action is pernicious and fearful in proportion to the organic and saline matters which their waters hold in solution; hence the reason why salt marshes and those near the sea-coast are peculiarly injurious to health. The drying up or submersion of marshes present conditions analogous to a galvanic pile deprived of moisture or overflowed, the effects of which are null or very trifling.

3d. The works of naturalists and physiologists have demonstrated that the electricity produced by our machines exerts a special influence upon the nervous system; experience and close observation of facts prove that the diseases developed in a marshy atmosphere are always primarily nervous; and when they become inflammatory it is always by the reaction of the nervous system upon the heart and blood vessels that local and general phlegmasiæ are produced.

4th. The neuroses and intermittent fevers being occasioned, not by the action of a miasm that has never been detected either in the air or in the water of marshes, but through the influence of the exaggerated electricity, any means by which this morbid influence can be modified must naturally and reasonably be the best,

5th. Electric isolation happily fulfils this indication. This isolation may be obtained by fixing to the bed-steads, sofas and chairs, glass or resinous feet. A large number of observations have proven to me that all the patients thus isolated have been cured or relieved of distressing diseases, many of which have resisted all other known means.

The striking analogy between marshes and the galvanic pile, the nature of the affections produced under the influence of atmospheric and terrestial electricity, and the method of combatting them by isolation, leads us therefore, naturally to the conclusion that not only the diseases of which we speak, but all those which appear epidemically and whose etiology is unknown, are to be attributed to an exaggeration of general electricity, the intensity of which must produce those varied electro-magnetic conditions which disturb the harmony so necessary to the continuance of human health.—Translated from Gazette Medicale de Paris for the *South. Med. & Sur. Jour.*

